# Detection of fibular rotational changes in cone beam CT: experimental study in a specimen model

**DOI:** 10.1186/s12880-022-00913-3

**Published:** 2022-10-19

**Authors:** Nils Beisemann, Antonella M. Tilk, Jula Gierse, Paul A. Grützner, Jochen Franke, Jeffrey H. Siewerdsen, Sven Y. Vetter

**Affiliations:** 1 MINTOS-Medical Imaging and Navigation in Trauma and Orthopaedic Surgery, BG Trauma Center Ludwigshafen at Heidelberg University Hospital, Ludwig-Guttmann-Str. 13, 67071 Ludwigshafen, Germany; 2 University of Texas MD Anderson Cancer Center, Houston, TX USA

**Keywords:** 3D-imaging, Ankle, Cone beam CT, Fibular rotation, Syndesmosis, Malposition

## Abstract

**Background:**

In syndesmotic injuries, incorrect reduction leads to early arthrosis of the ankle joint. Being able to analyze the reduction result is therefore crucial for obtaining an anatomical reduction. Several studies that assess fibular rotation in the incisura have already been published. The aim of the study was to validate measurement methods that use cone beam computed tomography imaging to detect rotational malpositions of the fibula in a standardized specimen model.

**Methods:**

An artificial Maisonneuve injury was created on 16 pairs of fresh-frozen lower legs. Using a stable instrument, rotational malpositions of 5, 10, and 15° internal and external rotation were generated. For each malposition of the fibula, a cone beam computed tomography scan was performed. Subsequently, the malpositions were measured and statistically evaluated with t-tests using two measuring methods: angle (γ) at 10 mm proximal to the tibial joint line and the angle (δ) at 6 mm distal to the talar joint line.

**Results:**

Rotational malpositions of ≥ 10° could be reliably displayed in the 3D images using the measuring method with angle δ. For angle γ significant results could only be displayed for an external rotation malposition of 15°.

**Conclusions:**

Clinically relevant rotational malpositions of the fibula in comparison with an uninjured contralateral side can be reliably detected using intraoperative 3D imaging with a C-arm cone beam computed tomography. This may allow surgeons to achieve better reduction of fibular malpositions in the incisura tibiofibularis.

## Background

Fractures of the ankle joint are a common entity. Nearly 9% of all fractures of the human body affect the ankle joint. The yearly incidence is about 180 out of 100,000 people [1–3]. Almost 11% of all ankle fractures are accompanied by an unstable syndesmotic injury [4, 5]. Primary goal of the operative treatment is to restore the anatomical position of the ankle joint in to prevent a premature osteoarthritis [6–11]. In particular, an increased external rotation of the fibula in the incisura tibiofibularis of more than 5° may lead to an increased pressure and will negatively affect the cartilage [8].

Intraoperative 3D imaging with a cone beam computed tomography (CT, three-dimensional mobile C-arm) appears to be beneficial for detecting an implant malpositioning or fracture malreduction [4, 9, 12–17]. Intraoperative 3D imaging reduces the risk of misinterpretation due to overprojection of anatomical structures. Standard planes of the area of interest can be reconstructed in coronal, axial, and sagittal views. In addition to the elaborate plane depiction, 3D imaging offers a more detailed visualization, especially of joint surfaces [14]. The AO Foundation (Arbeitsgemeinschaft für Osteosythesefragen, Davos, Switzerland) strongly recommends 3D visualization after open reduction and fixation of unstable syndesmotic injuries to evaluate length, rotation, and position of the fibula in the incisura tibiofibularis [4, 18]. This can be performed with either intraoperative cone beam CT or postoperative CT scan [4, 19–22].

Several studies have used standardized measurement methods to analyze fibular rotation and syndesmotic distances. Although, anatomical variations still present a challenge in gathering reproducible data, a previous study demonstrated that a location 4–6 mm distal to the talar joint line in the axial plane is the most reliable location to assess fibula rotation [23, 24].

To the best of our knowledge, a study to validate rotational changes of the fibula in a specimen fracture model using a cone beam CT has not been executed yet. The aim of the present study was to validate the accuracy of intraoperative cone beam CT measurements by using a specimen fracture model to analyze and detect rotational malpositions of the fibula in the syndesmotic region with 3D imaging. We hypothesized that a malrotation of the fibula in a standardized specimen model can be detected with cone beam CT.

## Methods

The experimental set-up included a cone beam computed tomography flat panel 3D C-arm (Cios Spin, Siemens Healthineers, Erlangen, Germany). 16 pairs of fresh-frozen lower legs were exarticulated at the knee joint, leaving the ankle joint, syndesmotic region and the interosseus membrane completely intact. Each pair was randomly divided into a control leg and a model leg, which were used to conduct the unstable syndesmotic injury. Exclusion criteria of the specimen are listed in Table 1.

**Table 1:** Exclusion criteria

Exclusion criteria
Damaged tissue
Posttraumatic alterations to the ankle joint
Anatomical aberrations
Rheumatoid alterations
Age < 18 years
Prior surgeries of the lower leg

### Experimental set-up

**Fig. 1:**
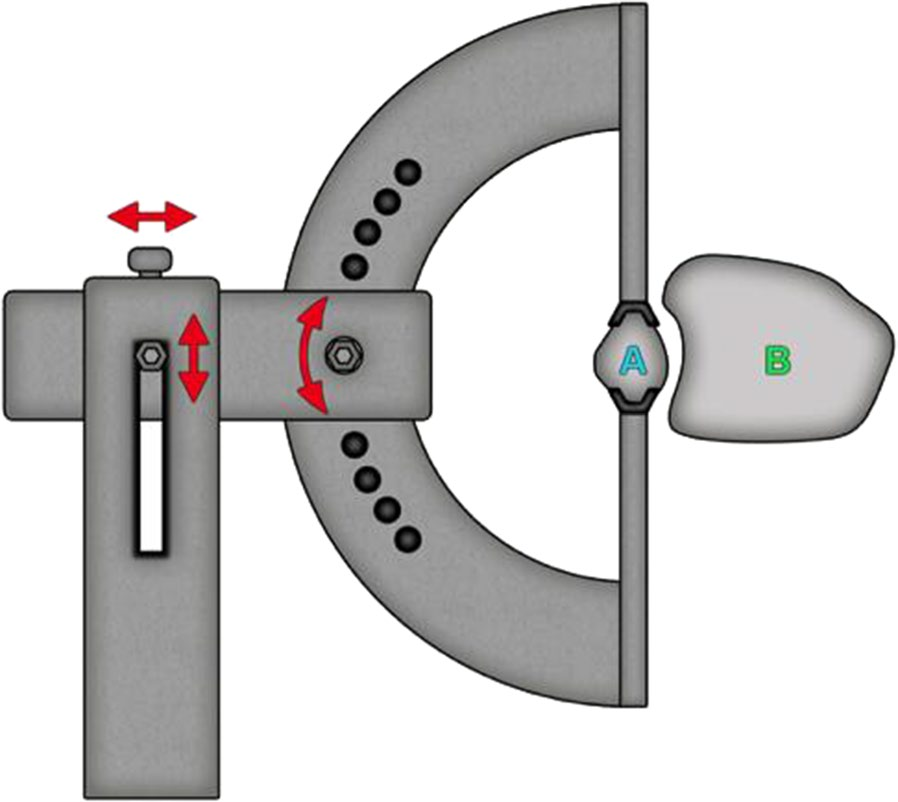
Schematic experimental set-up of the fibula (A) fixed in the rotation device. The jig was adjustable in height and lateral position, as well as rotation in 5° steps

First, a cone beam CT scan was taken of both ankles, to visualize the anatomy of the syndesmotic region of both uninjured ankle joints. A specially designed rotational device containing a semilunar grid and gripping pliers to fixate the fibula allowed the standardized rotation of the fibula in steps of 5° in each direction as shown in Fig. 1a. A lateral approach to the fibula was carried out and the anterior inferior part of the syndesmosis (anterior inferior tibiofibular ligament–AiTFL) with the tibiofibular joint exposed. All four ligaments of the syndesmosis (AiTFL–anterior inferior tibiofibular ligament, PiTFL–posterior inferior tibiofibular ligament, IOM–interosseus membrane, and TTFL–tibiofibulare transversum ligament) were dissected. The fibula was osteotomized 20 cm proximal to the tibiotalar joint line mimicking a Maisonneuve type fracture. Prior to this, the fibula was temporarily transfixed with a 2.0 mm K-Wire in the tibia, similar to a syndesmotic screw, to avoid shifts in the syndesmosis region.

**Fig. 2:**
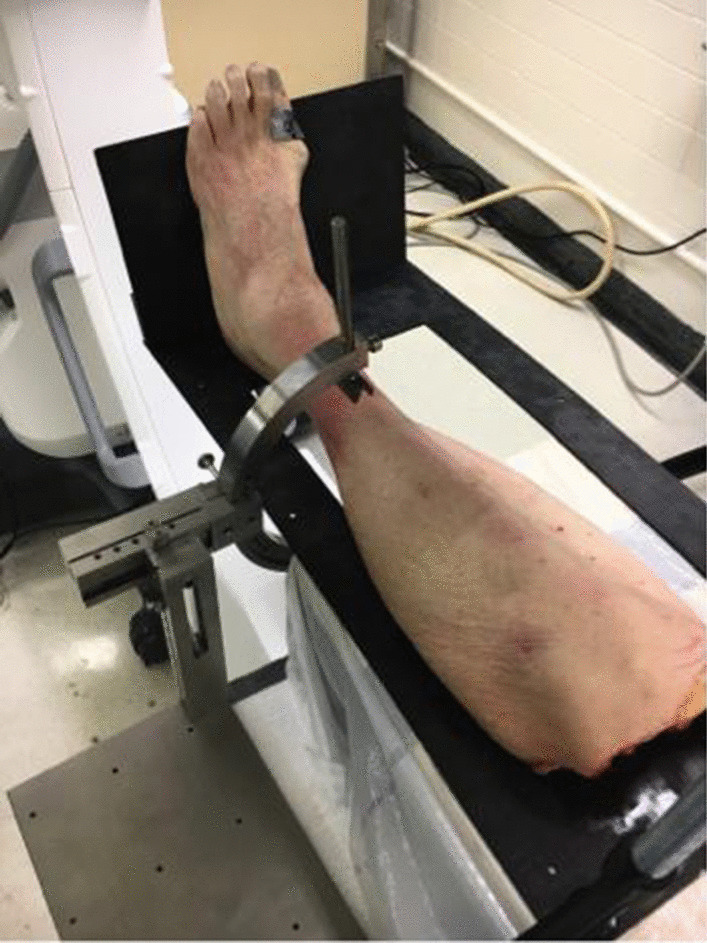
Rotational device with lower limb

All legs were placed on a radiolucent carbon fiber table as shown in Fig. 2. The rotational device fixated the fibula 6 cm proximal to the tibiotalar joint line with four screws and enabled positioning of the fibula at 5°, 10°, and 15° internal (IR) or external rotation (ER) in the incisura tibiofibularis. After removal of the K-wire transfixation, a 3D scan was conducted in each position: Anatomic, Fixated, 5° ER, 10° ER, 15° ER, 5° IR, 10° IR, and 15° IR. Due to similarities with the anatomic fibula position, the fixated fibula position was not taken into account for the statistical evaluation. Therefore, seven different fibula positions were analyzed: Anatomic, 5° ER, 10° ER, 15° ER, 5° IR, 10° IR, and 15° IR.

**Fig. 3:**
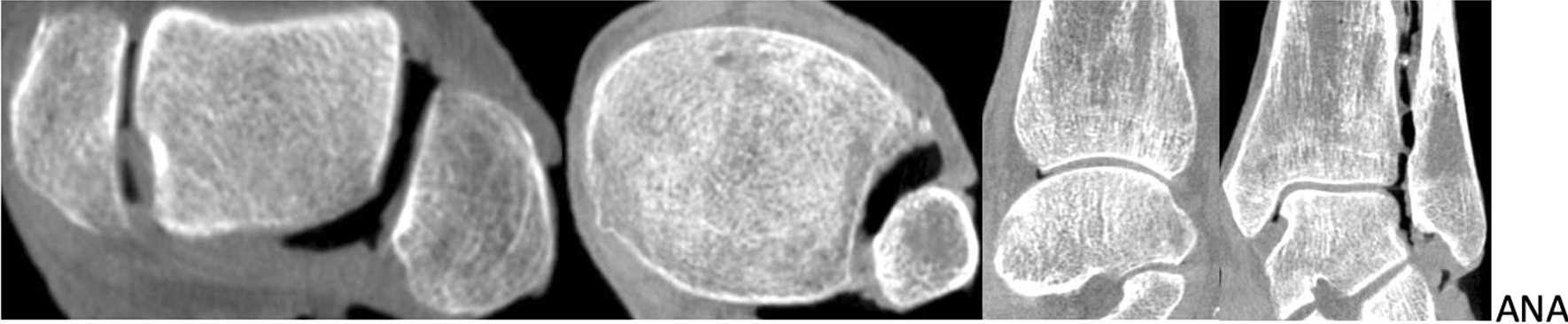
Anatomic position of the fibula from left to right: axial plane 6 mm distal of the joint, axial plane 10 mm proximal of the joint, saggital plane and coronal plane

**Fig. 4:**
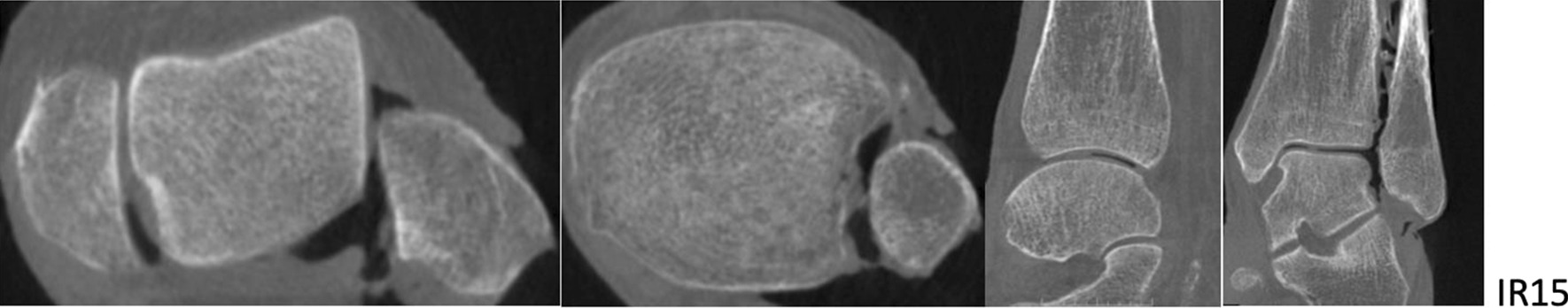
15° internal rotation of the fibula from left to right: axial plane 6 mm distal of the joint, axial plane 10 mm proximal of the joint, saggital plane and coronal plane

3D-imaging evaluation:

The angles parameters γ and δ (proximal to the tibial joint line and distal to the talar joint line), respectively, were determined in the axial plane using the DICOM viewer Horos (Horos Project 2020). γ was measured 10 mm proximal of the tibial joint line. Figures 3 and 4 show CBCT images of different fibula rotations.

**Fig. 5:**
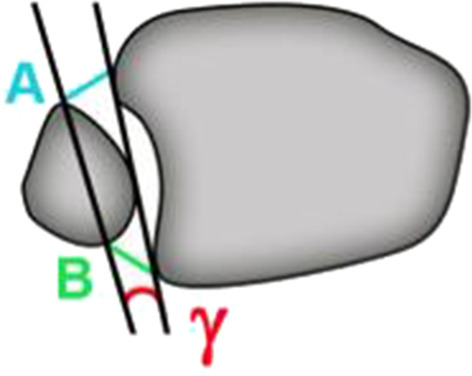
Measurement of angle γ at a location 10 mm proximal to the talar joint line

To evaluate the fibula rotation parameter γ was identified, which is the angle between the sagittal axis of the incisura tibiofibularis and the fibula, as shown in Fig. 5.

**Fig. 6:**
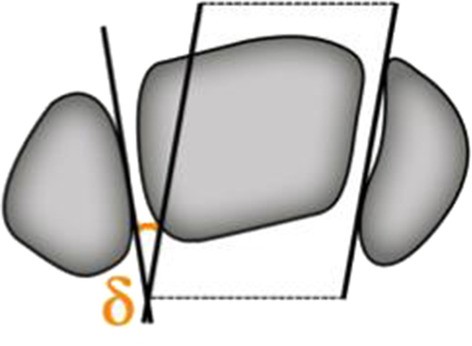
Schematic drawing of the measurement of angle δ at a location 6 mm distal of the talar joint line

Angle δ was also measured in the axial plane though, unlike the other parameters, the location selected for this measurement was found 6 mm distal of the talar joint line (Fig. 6).

All data sets were anonymized and randomly sorted. Each parameter was measured by one experienced surgeon, who was blinded for the fibular rotation applied. One lower leg pair had to be excluded because the 3D image dataset could not be reconstructed due to data readout errors.

In total 112 measurements were made for gamma and delta each.

The statistical analysis was performed using JMP software (SAS Institute, Cary, North Carolina, USA version 14.2.0). The mean and standard deviation for both angles were calculated and every pair student’s t-test evaluations performed. The mean values of the measured angles in 3D imaging were compared for the different fibula positions by applying student’s t-tests for each of the 21 pairs: ANA, 5° ER, 10° ER, 15° ER, 5° IR, 10° IR, 15° IR.

## Results

A total of 16 lower leg pairs were analyzed.

**Fig. 7:**
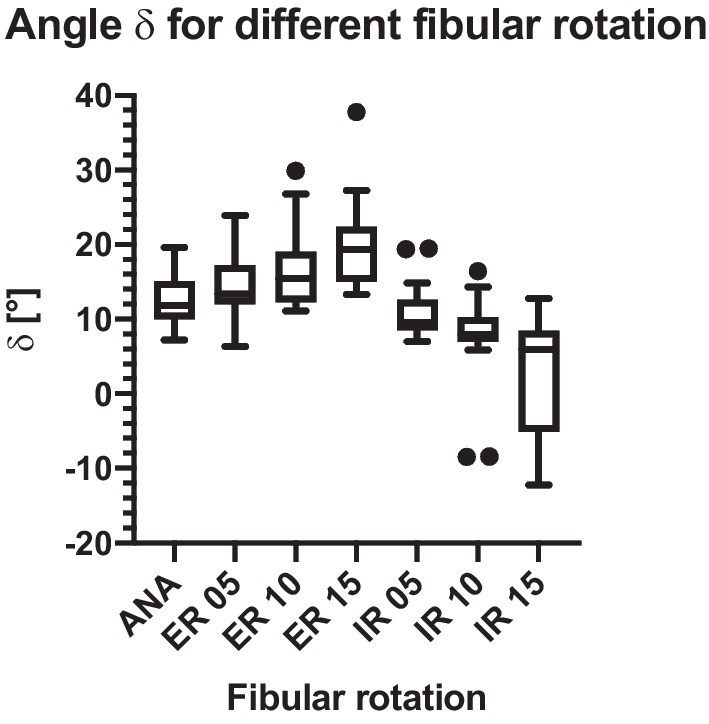
Angle δ mean and standard deviation

**Fig. 8:**
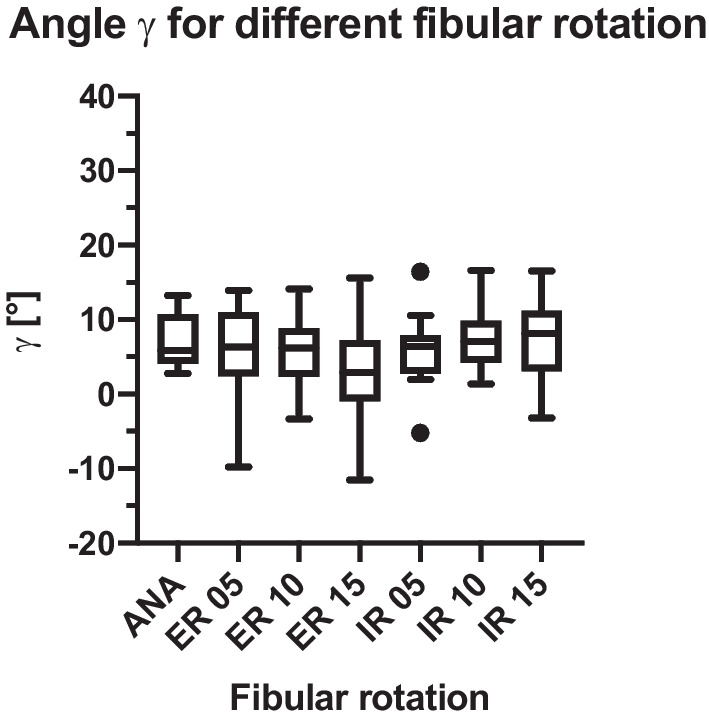
Angle γ mean and standard deviation

**Fig. 9:**
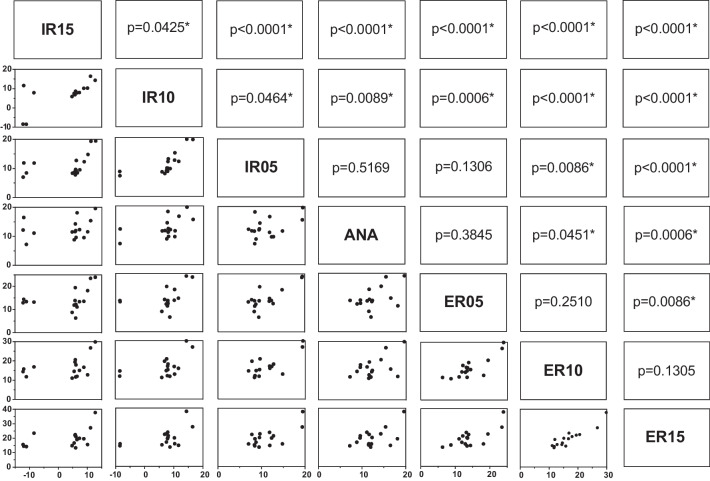
Pair t-test of parameter δ

Figures 7 and 8 show the mean values of δ and γ as well as the standard deviation measured in the cone beam CT scan. For angle δ (Figs. 7 and 9) a significant difference between the uninjured ankle (ANA) and an IR 10° (p = 0.009), IR 15° (p < 0.001), ER 10° (p = 0.045), and ER 15° (p < 0.001) could be detected. No significant difference was found for ANA vs. IR 5° (p = 0.517) and ANA vs. ER 5° (p = 0.385).

**Fig. 10:**
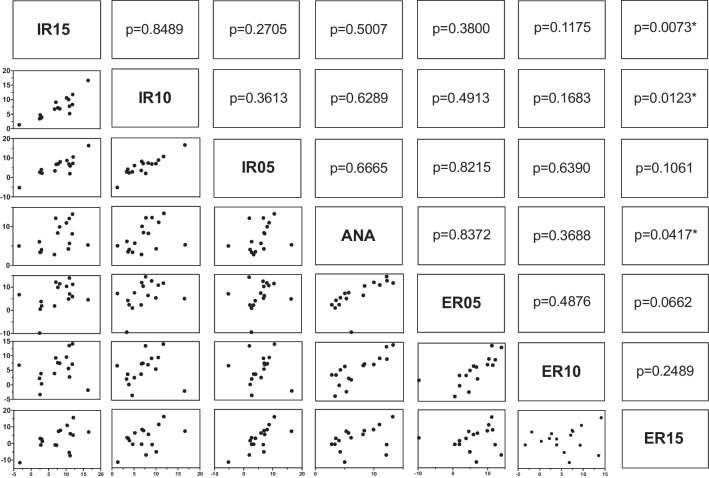
Pair t-test of parameter γ

Angle γ showed a significant difference for ANA vs. ER 15° (p = 0.04; Fig. 10). Yet, no significant difference was found for the comparison of the anatomical position with ER 10° (p = 0.369), ER 5° (p = 0.837), IR 5° (p = 0.667), IR 10° (p = 0.629) and IR 15° (p = 0.501).

## Discussion

Our hypothesis was that a malrotation of the fibula can be detected in a standardized specimen model with a cone beam CT measurement of the angles γ and δ.

Angle δ showed significant results for the comparison of ANA vs. ER 15°, ANA vs. ER 10°, ANA vs. IR 15°, and ANA vs. IR 10°, as well as several rotational differences between different non-anatomical positions. Therefore, we conclude that using cone beam CT, rotational deviations of the distal fibula can be assessed with the current measurement method of determining parameter δ for alterations of ≥ 10°. Deviations of 5° tended to show a difference but this was not statistically significant for ANA vs. IR 5° and ANA vs. ER 5°.

Parameter γ did reveal significant differences between ANA and ER 15°, the differences between all other clinically relevant combinations were not statistically significant.

The importance of an accurate syndesmotic reduction has already been described. Rotational malreduction can lead to instability, inferior function, and degenerative alteration due to changed biomechanics of the ankle joint [25–27]. Vasarhelyi et al. could show this clinical relevance based on the AOFAS score in their study on the detection of fibular torsion deformities after ankle fracture surgery using a novel CT method [25]. These results support the need for an accurate measurement method to detect rotational malreduction. Vasarhelyi et al. compared three different CT-based measurement methods in their study that compared the injured ankle with the uninjured ankle of the contralateral side. Two of the three CT-based methods showed a greater clinical relevance compared to the third one. In Method 1, described as the most adequate for the calculation of fibular torsion and asymmetry, the proximal rotation angle was measured at the caput fibulae by combining the medial tangent of the fibula with the horizontal base and determining the difference compared to the distal rotation angle, which was measured distal of the talar joint line by combining the medial tangent of the lateral malleolus with the horizontal base. Method 2 used the tangent of the medial fibular and the tangent of the incisura tibiofibularis at the level of the incisura to form an angle with a dorsal opening and was compared to that of the contralateral side. The fractures considered ranged from Weber B-C and included Maisonneuve fractures, whereas our study only included Maisonneuve fractures with a specimen model. However, Method 2, in particular, is similar to the described angle γ. It supports the thesis, in contrast to our findings, that Method 2 can be adequately utilized to detect fibular malrotation. The distal angle determined in Method 1 shows similarities to our measured angle δ and further backs our hypothesis on the most convenient location for measuring fibula rotation being distal to the talar joint line [24].

For angle δ, the t-test evaluations showed significant differences in the comparison of the mean anatomical position and 15° ER, 15° IR, 10° ER, and 10° IR. For angle γ, the t-test evaluations only showed a significant difference between the anatomical position and 15° ER. Prior et al. tried to establish a very similar way of measuring fibula rotation using the fibula axis (angle γ in our study). Due to the individual anatomical variation of the fibula, the authors were unable to reproduce reliable results and concluded that the malposition should be compared with the uninjured side [28]. Our method used the medial malleolar joint line and compared it with the uninjured side measuring a reliable angle (angle δ) distal of the joint line to evaluate the fibula rotation [24]. Summers et al. also used the uninjured ankle as a template comparison, as well as the talar dome, to evaluate syndesmotic reduction, supporting our technique in using angle δ in conventional fluoroscopy. The authors stated that the mortise view and talar dome lateral view in conventional fluoroscopy give enough information to evaluate syndesmotic reduction, making an intraoperative CT only necessary in some cases. However, this conclusion cannot be supported by prior results [29]. It seems more clinically adequate to analyze the fibula position intraoperatively using 3D imaging and to instantly compare the injured and uninjured ankle to achieve quality assurance [4, 14, 24, 25, 30, 31].

The absolute malposition produced by the malpositioning device could not be recognized, which means that the indication of a malposition given by these radiological parameters through cone beam CT imaging should always be considered in combination with the clinical condition and the overall coherence of the ankle in all planes.

This study compared two parameters (angle δ and γ) of fibula rotation in a standardized specimen model with the direct evaluation method of cone beam CT 3D imaging. A limitation to this study is that only specimen legs were used. In addition, the osteotomy was artificially produced just like the ligamentous tears of the syndesmosis and the interosseus membrane. Also, the rotation device has not been used in other studies, which limits the comparability of our results with results from similar studies. During the tests it became apparent that it is very difficult to consistently find the same measuring plane again and to take the same measuring points compared to the initial anatomical situation. Deviations from the original measuring points lead to a certain error in the angle measured.

The absolute angles measured suggest the fibula rotates less than the angle set in the jig. In this analysis, however, we considered measurements below the syndesmosis, so torsion forces in the fibula might reduce rotation in the fibula in the position set. The angle γ therefore seems more convenient for the rotation measurement due to its location directly at the syndesmosis, which was not consistent with the results presented above. Clinically, it seems the more significant measurement of angle δ might underestimate the actual rotational deviation in the syndesmotic region.

The potential advantages of intraoperative 3D assessment of fibula rotation and thus the chance of a more accurate reduction, have to be weighed against additional radiation exposure for patient compared to standard fluoroscopy based procedures. Furthermore, the acquisition costs for 3D CBCTs compared to a 2D imaging device has to be taken into account and weighted against a potentially reduced number of postoperative revisions [4, 16].

## Conclusions

In summary, this study shows that rotational deviations of ≥ 10° can be measured in 3D imaging using the parameter angle δ when compared to the contralateral uninjured side. Rotational deviations can therefore be detected and the reposition of the fibula clarified, which in turn should lead to a reduction of fibular malpositions in the incisura tibiofibularis.

## Data Availability

The datasets used and/or analysed during the current study are available from the corresponding author on reasonable request.

## Abbreviations

CT: Computed tomography
AiTFL: Anterior inferior tibiofibular ligament
PiTFL: Posterior inferior tibiofibular ligament
IOM: Interosseus membrane
TTFL: Tibiofibulare transverse ligament
ANA: Anatomical
IR: Internal rotation
ER: External rotation
